# Analyzing the Influence of Conservation Tillage and Manure on Soil Parameter Modulations in Croplands

**DOI:** 10.3390/plants13050607

**Published:** 2024-02-23

**Authors:** Ivan Dugan, Paulo Pereira, Ivica Kisic, Manuel Matisic, Igor Bogunovic

**Affiliations:** 1 Faculty of Agriculture, University of Zagreb, Svetosimunska 25, 10000 Zagreb, Croatia; idugan@agr.hr (I.D.); ikisic@agr.hr (I.K.); mmatisic@agr.hr (M.M.); 2 Environmental Management Laboratory, Mykolas Romeris University, LT-08303 Vilnius, Lithuania; paulo@mruni.eu

**Keywords:** tillage systems, crop yield, hydrological response, organic amendment, soil properties

## Abstract

Nowadays, when the human impact on the environment becomes prominent daily, specific steps are needed to mitigate or halt those changes. By far, agricultural land is most affected by the degradation process, leading to soil erosion and decreased soil quality. Sustainable measures are needed to find a solution to that problem. This study, located in an agricultural area in northwestern Croatia, gives an insight into how different tillage systems (conventional and conservation) with the addition of manure will affect soil physicochemical properties, hydrology response, and overall yield. To assess hydrological response, a rainfall simulator was used; meanwhile, soil samples were taken to determine bulk density, soil water content, water-stable aggregates, and soil organic matter. Soil water content did not show significant differences, whereas bulk density and penetration resistance yielded significantly higher values at 15–30 cm depth compared to 0–15 cm depth. Also, the conservation manure recorded lower bulk density and penetration resistance values than conventional tilled treatments. Water-stable aggregates and soil organic matter were severely affected by manure addition and yielded an increase after harvest. Hydrological response was delayed for the treatments with manure addition. Crop yield was also significantly higher for the conventional treatment with manure addition, whereas the control plots had lower yields. The results of this study show the positive aspects of conservation tillage, especially with manure addition, where soil quality is preserved or even increased.

## 1. Introduction

In pursuing a sustainable approach to agriculture, the pressing concern of soil degradation takes center stage, particularly in cropland soil erosion. Conventional tillage contributes significantly to soil erosion, compromising the soil’s structural integrity. Soil erosion is the primary source of soil and water losses, which can decrease organic matter and nutrient content and reduce water availability, thus endangering crop production [1,2,3]. High erosion rates will make the plow layer thinner, thus causing a decline in soil fertility and root development and, consequently, increasing the costs of fertilizer inputs. Crops are the leading protective agent that can prevent raindrop impact on soil and the surface and particle detachment process. Still, they cover the surface during part of the vegetation season, exposing the soil in developing stages to erosive rainfall events. The more the crops progress, the greater the surface cover will be. The rain will thus not directly affect the soil surface, as it will slowly run off the leaves onto the soil surface, thus enabling higher erosion events. Kisic et al. [4] state that during seedbed preparation, the soil is the most vulnerable to soil erosion, especially in maize cropping systems, where in just 60 days after sowing, around 80–90% of yearly soil erosion rates occur. In conventional agricultural systems, practices like monoculture and plowing are widespread. Even though plowing breaks soil compaction, integrates soil organic matter (SOM), enhances nutrient availability, and temporarily improves aeration, water infiltration, and drainage [5,6], overreliance on plowing accelerates organic matter breakdown, deteriorating soil structure, impending water movement and root penetration [7,8,9], and influencing the hydrological response of the ecosystem and potentially compromising water resources [10]. All the aforementioned tillage-induced degradation consequences negatively affect overall yield [11,12]. Several solutions can minimize this problem, starting with different tillage systems, soil amendment use, and mulching or cover cropping [13,14,15,16]. In contrast to plowing, conservation tillage is a suitable solution, as it enables residue retention, enhances organic matter content, maintains solid soil structure [17,18,19], and prevents erosion and nutrient runoff [20,21] while simultaneously enhancing water use efficiency in the agroecosystem. The anticipated positive impacts on soil quality include improved soil structure, increased organic matter content, and improved soil fertility, positioning conservation tillage as a key factor in mitigating soil erosion degradation and enhancing agricultural productivity in long-term periods [22,23,24,25]. Although different tillage systems have generated solutions to improve and preserve soil quality, adding organic amendments such as manure has had numerous positive effects. Farmyard manure (FYM) is rich in essential nutrients and organic matter and is an excellent solution for replenishing soil fertility and increasing microbial activity. In conventional tillage systems, FYM incorporation replenishes lost organic matter and essential nutrients [26,27], changing the soil structure and air-to-water ratio and increasing microbial activity [28,29,30]. In conservation tillage systems, FYM addition contributes to sustainable soil management and reduces soil degradation, which is highly recommended by the EU Commission [31,32]. Even though FYM incorporation is difficult in conservation tillage because of low soil exposure and mixing, it still has a vital role as a soil cover, maintaining soil structure, promoting water conservation, and reducing soil erosion [14,33,34]. Overall, conservation tillage and FYM application synergize, balancing soil conservation and nutrient enrichment [35]. The interaction between tillage and FYM addition practices underscores the intricate relationship between organic inputs and soil management strategies, influencing soil quality, structure, and productivity in conventional and conservation cropping systems. This study embarks on a comprehensive exploration to address the challenge of soil degradation by closely examining the influence of conservation tillage and manure on soil quality properties in rain-fed croplands. Since rain-fed croplands depend on natural precipitation, their susceptibility to climate variations is heightened. In this context, the present study will integrate rainfall simulation assessments to gauge the resilience of conservation tillage and manure-amended soils under determined precipitation scenarios. Evolving climate patterns that are more unpredictable and often extreme highlight the need to find appropriate sustainable agricultural practices that will adapt to these changing conditions. The objectives of this study are to (1) quantify and compare soil hydrological response and infiltration rate under different tillage systems and manure applications, (2) investigate soil physicochemical properties to understand the influence of different cropping systems, and (3) synthesize findings to offer a holistic understanding of the interconnected effects of tillage and manure addition on soil properties and crop yield.

## 2. Results

### 2.1. Soil Physicochemical Properties

The factorial ANOVA showed no significant differences between the treatments, depth, and time for soil water content (SWC) (Table 1). The SWC values for the depth 0–15 cm depth in emergence ranged from 40.4% for the conservation control treatment to 43.8% for the conventional manure treatment. After harvesting, higher SWC values were noticed for the conservation control (42.6%) and lower values were noticed for the conservation manure treatment (39.9%). At 15–30 cm depth in emergence, lower SWC values were recorded for the conservation control (39.5%) and reached 41.9% for the conventional manure treatment. The same pattern was observed for SWC values after harvest, with higher values observed for conventional manure (40.8%) and lower values observed for conservation control (39.5%). As for the bulk density (BD) values, no significant differences were noticed between sampling periods, whereas significant differences were noticed between treatments after harvest at 15–30 cm depth. At 0–15 cm depth in emergence, higher values were noticed for the conventional manure (1.31 g cm^−3^), whereas lower values were observed for conservation manure (1.27 g cm^−3^). The same pattern followed for the after-harvest measurement, so conservation manure (1.29 g cm^−3^) had lower and conventional manure (1.34 g cm^−3^) had higher BD values (Table 1). BD in emergence at 15–30 cm depth ranged from 1.31 g cm^−3^ for the conservation manure treatment to 1.38 g cm^−3^ for the conventional manure treatment. At the same depth after harvest, significant differences were noticed, where higher BD values occurred for the conventional manure (1.39 g cm^−3^) treatment compared to the conservation control (1.32 g cm^−3^) and conservation manure treatment (1.33 g cm^−3^). The conventional control (1.36 g cm^−3^) treatment did not significantly differ from the others (Table 1). In emergence, only significant depth changes at 0–15 cm and 15–30 cm were noted, whereas conventional manure recorded lower BD values at 0–15 cm depth (1.31 g cm^−3^) compared to 15–30 cm depth (1.38 g cm^−3^).

Table 1 also shows no significant differences between time and treatment for penetration resistance (PR), but certain changes were noticed between the two depths. At 0–15 cm depth in emergence, the PR values ranged from 0.64 MPa for the conservation manure to 0.89 MPa for the conservation control treatment. After harvest, lower values were recorded for the conventional manure and conservation manure (0.72 MPa and 0.73 MPa), whereas higher ones were recorded for the conservation control treatment (1.10 MPa). Meanwhile, at 15–30 cm depth, higher values in emergence were recorded for the conventional control (1.45 MPa), and lower PR values were recorded for the conventional and conservation manure treatments (1.07 MPa and 1.10 MPa). After harvest, the PR values ranged from 1.08 MPa for the conservation manure to 1.47 MPa for the conservation control treatment. Significant differences were noticed between the two depths for the conventional control and the conservation manure in emergence, with higher PR values observed at higher depths. The same pattern continued for the PR values for the conventional manure treatment, where 15–30 cm depth after harvest yielded higher values than 0–15 cm depth (Table 1).

In emergence, water-stable aggregates (WSA) showed no significant differences between treatments, ranging from 50.35% for the conventional manure treatment to 62.85% for the conventional control treatment. After harvest, significantly lower WSA was observed for the control conventional treatment (49.25%) compared to the manure conservation treatment (73.90%), whereas the conventional manure treatment (67.94%) and the control conservation treatment (63.90%) did not statistically differ from the others (Table 2). In emergence, the conventional control treatment had significantly higher WSA values compared to after harvest, whereas the conventional manure treatment recorded statistically higher values after harvest. The conservation treatments did not significantly differ between the two periods, whereas higher WSA values were noticed after harvesting. Before emergence, soil organic matter did not differ between treatments, with values ranging from 3.35% to 3.47%. After harvest, significantly higher SOM values were recorded for the conventional manure treatment (3.77%) compared to the conventional control treatment (3.12%), whereas the conservation treatments (3.47%, 3.46%) did not significantly differ from those two. The ANOVA analysis showed no SOM value differences for treatments between the two investigated periods. 

### 2.2. Hydrological Response

**Figure 1:**
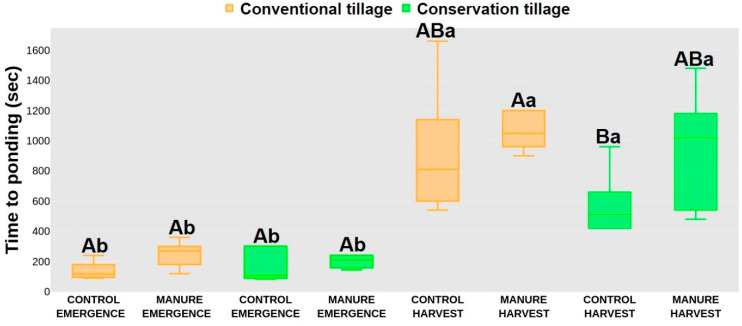
Time to ponding. Different letters represent significant differences at *p* < 0.05. Capital letters indicate statistical differences between treatments; lowercase letters indicate statistical differences between seasons. Data were collected in May (emergence) and October (harvest) 2021 at a field close to Marija Magdalena, northwestern Croatia.

**Figure 2:**
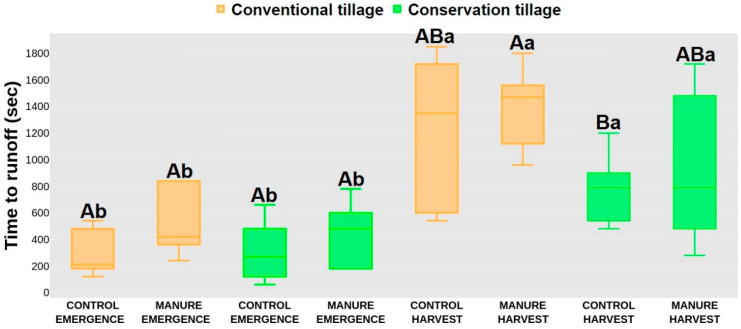
Time to runoff. Different letters represent significant differences at *p* < 0.05. Capital letters indicate statistical differences between treatments; lowercase letters indicate statistical differences between seasons. Data were collected in May (emergence) and October (harvest) 2021 at a field close to Marija Magdalena, northwestern Croatia.

**Figure 3:**
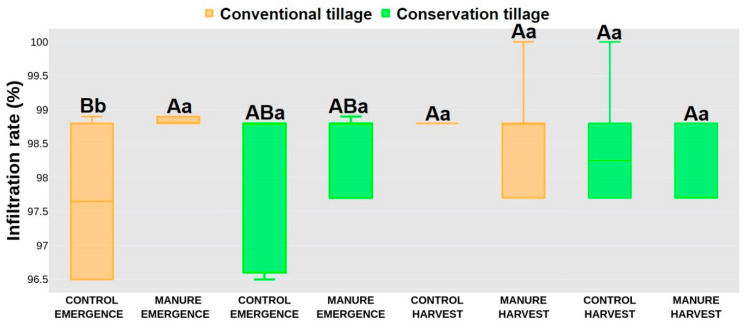
Infiltration rate. Different letters represent significant differences at *p* < 0.05. Capital letters indicate statistical differences between treatments; lowercase letters indicate statistical differences between seasons. Data were collected in May (emergence) and October (harvest) 2021 at a field close to Marija Magdalena, northwestern Croatia.

In the emergence period, time to ponding (TP) did not show significant differences between treatments, with higher values were recorded for the manure-amended treatments (250 s, 201 s) compared to the control treatments (140 s, 165 s). After harvest, significantly lower TP values were recorded for the conservation control treatment (580 s) compared to the conventional manure treatment (1060 s), whereas the conventional control (927 s) and conservation manure (953 s) treatments did not statistically differ from the conventional manure and conservation control treatments (Figure 1). Between seasons, higher TP values were recorded for all treatments after harvest. A similar trend for the TP values was observed for time to runoff (TR) in emergence, where lower values were also recorded for the control treatments (290 s, 310 s), independently of the tillage systems, compared to the manure treatments (520 s, 450 s). After harvest, significantly lower values were recorded for the conservation control treatment (783 s) compared to the conventional manure treatment (1398 s), whereas the conventional control and conservation manure treatments did not statistically differ from those with TR values of 1235 s and 923 s. After harvest, the conventional treatments recorded higher TR values than in the emergence period. The conservation treatment did not show significant differences between periods, even though the TR values were greater after harvest (Figure 2). The infiltration rate (IR) in emergence yielded significantly higher rates for the conventional manure treatment (98.8%) compared to the conventional control treatment (97.7%), whereas the conservation treatments (98.1%, 98.5%) did not statistically differ from them. After harvest, no statistical differences were noted between treatments. Also, there were no differences between periods for each treatment, except for the conventional control (99.8%), where significantly higher values were recorded after harvest (Figure 3).

### 2.3. Crop Yield

**Figure 4:**
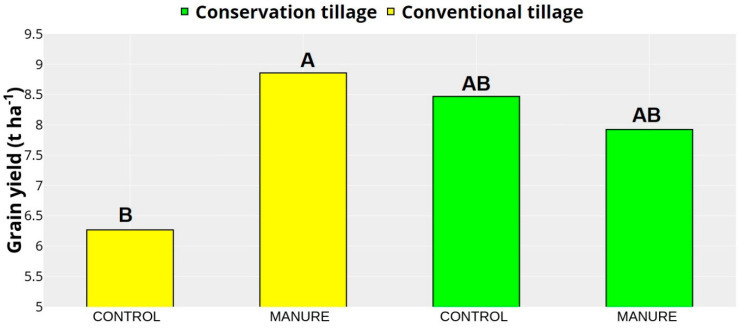
Grain yield. Different letters represent significant differences at *p* < 0.05. Capital letters indicate statistical differences between treatments. Data were collected in May (emergence) and October (harvest) 2021 at a field close to Marija Magdalena, northwestern Croatia.

The maize yield presented in Figure 4 significantly differed amongst treatments, with a higher yield observed for the conventional manure treatment (8.86 t ha^−1^) compared to the conventional control treatment (6.27 t ha^−1^). In comparison, the treatments under conservation tillage systems did not statistically differ from those under conventional tillage systems (8.47 t ha^−1^, 7.92 t ha^−1^).

## 3. Discussion

### 3.1. Soil Properties

The SWC did not significantly differ between treatments and depths. Such results are possibly because of (1) specific local climate conditions; (2) soil type and texture, as clayey soil tends to retain water for longer periods, thus masking the short-term effect of established treatments; and (3) the short-term study duration—since soil water dynamics are usually gradual, the short period of investigation may not have captured the full range of effects that tillage and manure can have on SWC. The BD showed a slight increase in both depths after harvest compared to emergence. Such values were expected since the soil had compacted naturally with time after seeding and because of machinery trafficking [36] through the vegetation season. It seems that manure addition did not have a crucial impact on soil BD in either tillage management. Even though conventional tillage makes suitable seedbeds for planting and is efficient for weed control, such tillage will lead to soil compaction in the long term. Constant soil structure disruption and compaction caused by heavy machinery results in higher soil BD due to reduced pore space, limited water infiltration, root penetration, and overall soil aeration [37]. Conservation tillage, on the other hand, helps preserve soil structure and minimize soil compaction, as the presence of crop residues acts as a protective layer, preventing soil erosion and enhancing water infiltration and aggregate formation [38,39,40]. These factors combined contribute to lower soil BD. The present research was established in 2021, and the short experimental period from the date of establishment is very likely a reason for the absence of statistical justification of the soil compaction status between tillage treatments. However, conservation manure plots exhibited the lowest compaction, measured by BD and PR. PR recorded lower values for the treatments with manure addition and treatments under conservation tillage systems. Manure application has contrasting effects on PR at different depths. At a depth of 0–15 cm, the organic matter from manure improves soil structure, enhances aggregation, and reduces compaction, typically leading to lower PR [41,42,43,44]. At 15–30 cm depth, the impact of manure on PR can be less pronounced, and a stronger impact of manure on subsoil compaction depends on factors such as manure distribution, decomposition rates, and root ability to penetrate deeper layers. Usually, conventional tillage has a positive effect on the topsoil layer since frequent tillage initially decreases PR by breaking up compacted soil and promoting aeration [45]. On the subsurface layer, the effects of conventional tillage may be more variable, even though the immediate impact could be a reduction in PR due to soil disruption. Repeated tillage will contribute to compaction over the long term [46], whereas moldboard plowing often enhances plow pan creation [47]. On the other hand, conservation tillage generally leads to an increase in PR on the topsoil due to minimal soil disturbance and the retention of crop residues on the surface; their decomposition can create a layer of fine organic material that, when mixed with soil particles, may increase soil strength and resistance to penetration [48]. Meanwhile, on the subsoil, reduced soil disturbance allows for the maintenance of soil structure and aggregation, contributing to improved porosity and reduced compaction at greater depths [18,19]. The present results indicate a trend of decreased PR at plots with incorporated manure. At the same time, the values did not exceed critical levels (<2 MPa, according to [49]) for root development in either tillage system despite the timing of the measurements. Water-stable aggregates demonstrated significant differences between the treatments. Before emergence, the WSA values were significantly higher for the conventional control and conservation manure treatments, which is partly opposite to existing literature findings [10,43,50,51]. This is probably because of (1) the short period between experiment establishment, the application of FYM, and soil sampling, and (2) natural soil fertility, which varied significantly between the plots since the previous fertilization management was unfamiliar. However, after harvest, the WSA values reached our expectations, with manure addition and conservation practices playing a vital role in increasing aggregate stability. When manure is applied to the soil, it promotes the formation and stabilization of aggregates, as organic residues from manure enhance the binding of particles [52], thus creating larger and more stable aggregates. On the other hand, conservation tillage severely impacts WSA, with minimum soil disturbance leaving crop residues on the soil surface, thus reducing surface sealing and promoting stable aggregate formation [53]. Before emergence, there were no significant differences between the treatments for SOM, whereas after harvest, there was a slight increase in SOM for the conservation treatments. Significant differences after harvest were only noticed in the treatments under conventional tillage systems. The control plot recorded an SOM decrease due to (1) lack of fertilization, (2) intensive tillage exposing the soil to atmospheric conditions, and (3) erosion events that caused organic matter loss down the slope. Manure addition to the conventional tilled soil plays a vital role in increasing SOM and is a continuous source of energy for microorganisms [54]. Microbial activity breaks down organic residues from manure, contributing to the formation of stable SOM [55]. Conservation tillage has a similar effect, where the crop residues gradually decompose, thus adding organic matter to the soil [56]. Organic matter incorporation into topsoil enhances SOM content, fostering improved soil structure, water retention, and nutrient cycling. It is important to highlight the combined effect of manure and conservation tillage, from which soil quality will benefit through enhanced microbial activity, nutrient cycling, and the formation of stable aggregates, thereby improving overall soil structure and resilience [57,58].

### 3.2. Hydrology Response

The hydrology response in the present study was affected more by manure addition than by the tillage system. Even though conservation tillage minimizes soil disturbance, it promotes water infiltration [59]. With less disturbance to the topsoil layer, water can more readily infiltrate the soil profile, resulting in a longer period of time for ponds to form on the soil surface [60]. Also, conservation tillage accumulates residues—a protective layer that mitigates the impact of raindrops and minimizes soil erosion. This can lead to a longer TR, allowing for greater water absorption and reduced surface water flow. As for the infiltration, it is affected by enhanced soil structure and aggregate stability. Also, the surface cover helps to protect the soil from the impact of raindrops, preventing crusting and improving water absorption [61,62]. In contrast, conventional tillage involves more intensive soil disturbance, breaking up the soil structure and creating a fine seedbed. This reduces water infiltration and increases surface runoff, resulting in postponed ponding creation. The potential for soil erosion is also higher under conventional tillage, contributing to a shorter TR [63,64]. Frequent soil disturbance under a conventional tillage system will lead to soil compaction and decreased pore space, limiting water infiltration. The exposed bare soil surface is more susceptible to crusting, reducing infiltration rates and increasing the likelihood of surface runoff [6]. As mentioned before, manure application had a greater effect on soil hydrology in our study since manure generally enhances soil structure and organic matter content, improving water infiltration. Manure contributes to increased porosity, aggregate stability, and water holding capacity, facilitating efficient water movement through the soil profile. On the other hand, increased organic matter from manure addition helps to create a more stable soil surface, reducing the risk of crusting and erosion [65,66,67]. Such newly created conditions will favor postponed TP and TR, as in our research (Figure 1 and Figure 2). The control plots had significantly lower TP and TR, as the soil on those plots was not capable of efficiently absorbing and retaining water, thus leading to increased surface runoff. Although the infiltration rate did not show any significant differences between treatments, a higher IR was still observed for the treatments with applied manure, whose presence improved soil structure, thus preventing surface sealing and encouraging water to penetrate the soil [68,69]. On the other hand, the lower IR in the control plots was due to more compacted soil and sealed surface, which can lead to increased surface runoff. 

### 3.3. Grain Yield

Manure contributed to increased yields in both tillage systems. It can be seen that manure improved the soil’s physical environment through better soil structure (higher WSA) in both tillage systems and decreased resistance to rooting (measured by PR) in addition to the control plots. The BD did not follow this pattern, very likely due to the higher sensitivity of PR than BD in soil tillage–amendment studies, as has been documented before [49]. Moreover, sufficient manure addition significantly increases grain yield due to improved nutrient availability [70,71,72]. In our case, conventional tillage greatly impacted manure decomposition since it enabled the manure to be evenly mixed with soil, thus increasing organic matter decomposition and mineralization. This distributes the nutrients evenly through the maize root zone, thus directly affecting the grain yield [73,74], as in our research, where the grain yield was the highest for the treatment with manure addition and the conventional tillage system. On the other hand, lower yield was also recorded for the conventional tillage plots, but organic amendments were not added. This affected the grain yield, as presented in Figure 4, where conventional tillage, despite having a few advantages (e.g., soil aeration, better organic matter mixing into the soil), had more pronounced negative effects, where the soil from deeper layers were under the atmospheric conditions, directly affecting SOM decomposition and loss. In the long term, conventional tillage increases soil compaction, contributing to low root development and decreased yield [75,76,77]. The treatments under conservation tillage did not show significantly higher yields than the conventional ones but showed yield stability between the manure and control treatments. The actual effect of manure addition in the first year under conservation tillage may not appear due to the following reasons: (1) Low soil mixture slows down the organic matter breakdown process; (2) even though conservation tillage improves soil structure over time, the benefits may not be fully realized in the initial year since the crop and soil may not reach an optimal equilibrium; and (3) conservation tillage often results in increased weed pressure in the initial year, thus competing with crops for nutrients and water. Several studies indicate that conservation tillage will gradually increase yield or achieve equilibrium between conservation and conservation tillage after several years of consistent soil management [78,79,80,81]. The conservation tillage effect also depends on organic amendment addition, which can speed up those processes to satisfy the need for yield but also for soil quality. 

### 3.4. Implications for Management

The examination of soil physicochemical properties and hydrology response in this study provides valuable insight into the potential implications for agricultural management. Even though certain shortcomings are associated with a single-season focus on one specific crop, the detailed analysis of these soil attributes and grain yield information provides beneficial information for farmers and landowners. The assessment of soil properties such as BD, SWC, and PR offers a deeper understanding of the immediate effects of the manure addition combined with different tillage systems on soil characteristics. These insights can guide farmers to make informed decisions about soil preparation and management practices to optimize crop productivity. Since our experimental site is susceptible to water erosion, evaluating WSA was pertinent, as it shed light on the soil’s resistance to water-induced degradation. Combined with conventional and especially conservation tillage, manure addition contributes to developing erosion-control strategies [13,82]. Further, SOM assessment is integral to understanding the long-term effects of the studied treatments on soil quality. SOM is a crucial indicator for soil fertility, moisture retention, and overall ecosystem resilience [83,84,85]. The measured SOM levels offer a glimpse into the potential impacts of manure and tillage practices on soil organic carbon content, so a more in-depth exploration of SOM dynamics over multiple seasons would be essential for providing farmers with a comprehensive understanding of agroecosystem sustainability. The findings considered soil physicochemical properties, hydrological response, and crop yield and can guide land managers in optimizing their choices, potentially mitigating soil degradation. With acknowledgment of this study’s limitations, stakeholders can leverage this wealth of information to make informed decisions, tailoring agricultural practices to enhance soil quality and water stability and ensure crop productivity and overall ecosystem resilience in the face of evolving environmental challenges.

### 3.5. Study Limitations

Although this study presents a valuable investigation into the impacts of a combination of manure addition with different tillage systems, several limitations should be acknowledged to ensure a comprehensive understanding of the findings. First, this study’s focus is restricted to a single-year comparison, which can raise concerns about the generalizability of the results. Agricultural systems, as in our research, are inherently dynamic. The effects of manure addition and different tillage practices on maize growth and soil properties can vary across seasons due to climatic variations and seasonal-specific agronomic practices. Consequently, the findings may not fully capture the full range of potential outcomes, thus limiting the research’s ability to provide robust recommendations for year-round agricultural management. Furthermore, this study’s focus only on maize may limit the broader applicability of this study’s conclusions. Different crops exhibit diverse responses to tillage and manure application, and the specificity of the investigation to maize might not adequately represent the broader spectrum of crops cultivated in the region. Also, the incorporation of manure, tillage systems, and control plots can introduce another level of complexity to the research. While the multifactorial approach is commendable for capturing interactions between different variables, it can pose challenges in isolating the specific effects of each factor. Untangling the individual contributions of manure and various tillage systems to maize performance becomes intricate, potentially obscuring nuanced insight into the efficacy of each treatment. To address this limitation, future research may consider implementing a factorial experimental design, allowing for a more precise evaluation of manure and tillage’s independent and interactive effects on maize growth and soil properties. The study design should also account for the potential long-term impacts of manure application and tillage practices. Agricultural sustainability requires understanding the cumulative effects on soil quality and crop productivity over extended periods. A single-season study may not adequately capture the enduring consequences of the applied treatments, hindering the ability to make informed decisions about the long-term sustainability of the proposed agricultural practices. While this study provides valuable insight into the immediate effects of combining manure and tillage systems on maize fields, it is essential to address the limitations mentioned above so future research endeavors will contribute to a more holistic understanding of sustainable agricultural practices and facilitate the development of recommendations applicable across diverse agricultural contexts.

## 4. Materials and Methods

### 4.1. Study Area

**Figure 5:**
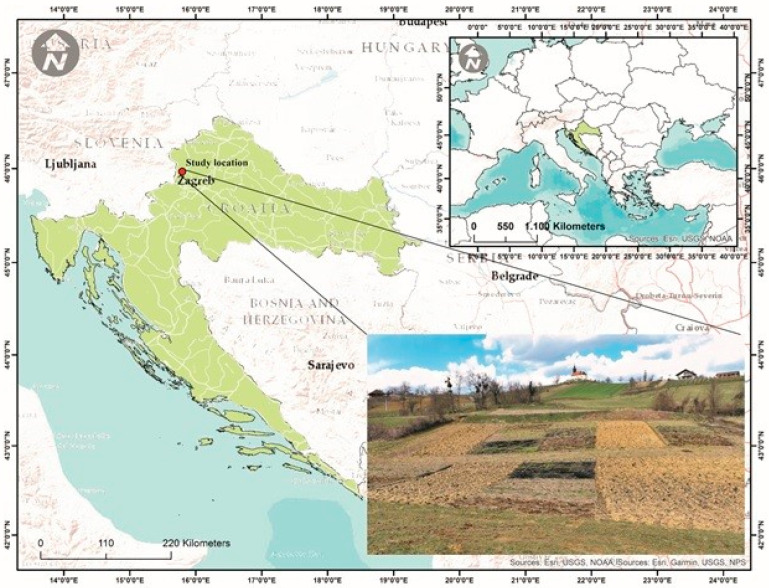
Location of the experimental site and view of the experimental plots in Marija Magdalena, Croatia.

**Figure 6:**
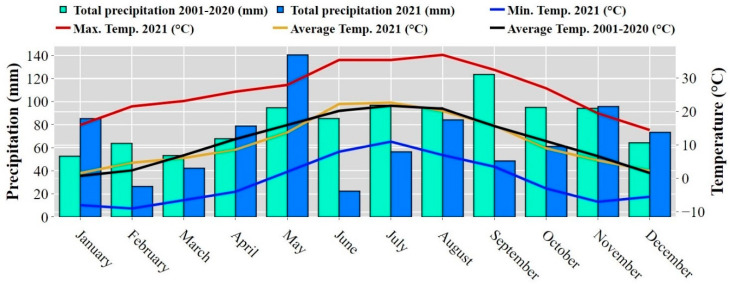
Monthly precipitation and temperature throughout the experimental period for a long-term period (2001–2020).

This study was carried out in northwestern Croatia in Marija Magdalena (45°55′ N; 15°44′ E) at an elevation of 211 m a.s.l. and an average inclination of 11° (Figure 5). The terrain is hilly, predominantly surrounded by croplands used for livestock farming, permanent plantations (vineyards, apple, and plum orchards), and forests. According to Köppen’s climate classification [86], the climate is Cfb, with warm summers. The mean annual precipitation (2021) was 815.6 mm, with a minimum of 26.5 mm in February and a maximum of 140.5 mm in May, while the mean annual temperature in 2021 was 11 °C, with lower temperatures indicated in January (1.6 °C) and higher temperatures indicated in July (22.7 °C). Also, precipitation and temperature averages (2001–2020) are presented in Figure 6. Meteorological data were collected from the nearby meteorological station (5 km away from the experimental site). The soil type is classified as Stagnosols [87]. The overall soil properties at different horizons are shown in Table 3.

### 4.2. Experimental Design

The study site was established in November 2020. Before the experiment was established, the usual crop rotation system was maize–winter wheat–alfalfa–maize–winter barley–maize. Before the tillage and manure application were made, the soil surface was covered with maize residues. The trial was set up as a split-plot design with tillage as the primary treatment and amendment application as a sub-treatment. Two different tillage systems, conventional and conservation tillage, were involved. Conventional tillage was conducted using a moldboard plow to 25 to 30 cm depth and a rotary harrow to 8 cm depth for seedbed preparation. Conservation tillage comprises non-invertive tillage to a 30 cm depth with a cultivator and seedbed preparation to a depth of 8 cm with a harrow. The primary tillage was performed in November 2020, while the secondary tillage was conducted in April before maize seeding. Detailed information about the characteristics of the equipment and tractor used are presented in Table 4. Two sub-treatments were established within the tillage treatments: farmyard manure (40 t ha^−1^) and control (no addition). Plot sizes were 8 × 6 m (48 m^2^) in size, and the measurements were conducted on 24 separate plots.

### 4.3. Fieldwork

Maize (Syngenta Infinite FAO 410, 75,000 seeds per hectare) was sown on 24 April 2021. Maize crop protection, herbicide use, and fertilization (300 kg ha^−1^ of NPK (15-15-15)) operations were performed equally on all treatments. Fieldwork considering sampling was carried out after emergence (May 2021) and after the maize harvest (October 2021). Soil core samples (96 in total) were collected at 0–15 cm and 15–30 cm (4 treatments × 2 depths × 2 sampling times × 6 replicates). Additional undisturbed soil samples were collected at 0–15 cm depth and stored in plastic boxes to determine WSA and SOM. Soil PR was measured by an electric hand-pushed cone penetrometer (Penetrologger, Eijkelkamp, Giesbeek, The Netherlands) using a cone with a 3 cm^2^ base area, a 60° included angle, and an 80 cm driving shaft. Infiltration and runoff generation properties were determined using rainfall simulation experiments on the same soil sampling date. Twenty-four rainfall simulations per season, 48 in total, were carried out to determine the hydrological properties of the investigated soil under different soil management. For this purpose, a rainfall simulator was used for half an hour of the rainfall simulation at an intensity of 58 mm h^−1^, as described in Bogunovic et al. [88]. Rainfall intensity was adjusted based on the time the nozzle (VeeJet 80/100 nozzle, pressure at 0.5 bar) remained at the reversal points and the nozzle turning speed [89]. The plots underneath the simulator were circular (metal ring of 1 m diameter), with a 0.785 m^2^ surface area, which was pressed up to 10 cm into the soil. TP and TR were measured using a chronometer during the simulations. The IR was calculated from rainfall simulation and runoff data based on the following equation:

(1) $$\mathrm{IR}=100-\left(\frac{m\;\mathrm{sediment}*\;\left(\frac{m\;\mathrm{water}}{m\;\mathrm{sample}}\right)}{V\;\mathrm{applied}\;\mathrm{water}}\right)\;*\;100$$

where IR is the percentage of infiltrated water during the rainfall simulation experiment, m sediment is the mass of sediment from the overland flow sample (g), m water is the mass of water from the overland flow sample (g), and m sample is the mass of collected overland flow sample (g). V applied water is the amount of water used during a rainfall simulation experiment over the catchment plot (mL).

A harvester determined maize yields using six passes per treatment. The grain was cleared, dried to 14% moisture content, and weighed to obtain the overall maize yield.

### 4.4. Laboratory Analysis

Soil BD and SWC were determined by weighing the samples before and after capillary wetting and drying at 105 °C for 48 h, after which the samples were calculated based on the gravimetric method: 

BD = dry sample/soil volume(2)

SWC = ((samples with filed water content − dry samples)/soil volume) × 100(3)

Undisturbed soil samples were gently hand-prepared as in Dıaz-Zorita et al. [90] to avoid the possibility of breaking down the formed aggregates, after which they were air dried at a room temperature of 25 °C for seven days, sieved, and weighted. The aggregate fraction from 1 to 2 mm was taken to determine aggregate stability using Eijkelkamp’s wet sieving apparatus and the method derived from Kemper and Rosenau [91]. The following formula yielded the percentage of WSA:

(4) $$\mathrm{WSA}=\frac{\mathrm{Wds}}{\mathrm{Wds}+\mathrm{Wdw}}$$

where WSA is the percentage of stable water aggregates, Wds is the weight of aggregates dispersed in dispersing solution (g), and Wdw is the weight of aggregates dispersed in distilled water (g).

The SOM was determined using the wet digestion method [92] after air-drying, milling, and sieving the soil samples through a 2 mm mesh. 

### 4.5. Statistical Analysis

Firstly, all the data were checked for normality with the Shapiro–Wilks test. Normal distribution of the data was considered at a *p* > 0.05. Water-stable aggregates, SOM, PR, and TR followed the Gaussian distribution, while BD, SWC, and crop yield data were square root and logarithmic-transformed to meet normality requirements. Factorial ANOVA analysis was applied, and if significant differences were identified at a *p* < 0.05, a post hoc Duncan’s test was performed. All the statistical analyses were carried out with the software Statistica 12.0 [93], while the graphs were created using Plotly [94]. All the data throughout the paper are presented in their original state. 

## 5. Conclusions

This study investigated the synergistic effects of manure addition and different tillage systems on soil properties, hydrology response, and maize yield. Non-significant post-harvest measurements revealed increased BD and PR as natural and machinery-induced re-compaction. Conservation tillage exhibited lower BD than conventional tillage, emphasizing its soil-preserving benefits. SOM and WSA increased after harvest in conservation treatments, reflecting the contributions of manure and conservation tillage to microbial activity and nutrient cycling. In spite of this study only lasting one year, we have already noticed a pronounced reduction in runoff generation with the incorporation of manure. The ensuing analysis of grain yield underscores the notable positive influence of manure, especially within conventional tillage systems. Despite study limitations, including the single-year focus and specific crop choice, the findings offer valuable insight for land managers and guiding considerations for sustainable soil quality, water stability, and crop productivity. Future investigations should consider these limitations for a more comprehensive understanding of the long-term effects of combined manure and tillage practices.

## Figures and Tables

**Table 1:** Results of factorial ANOVA analysis considering soil properties between seasons, depth, and treatments. Different letters after mean values in the columns represent significant differences at *p* < 0.05. Capital letters show statistical differences for treatment between two seasons; small letters show statistical differences between treatments at one depth in two seasons. The ^ns^ and * after letters represent the difference in treatment between two depths in the same period. Abbreviations: BD, bulk density; SWC, soil water content; PR, penetration resistance. Data were collected in May (emergence) and October (harvest) 2021 at a field close to Marija Magdalena, northwestern Croatia.

Depth	Time	Treatment	SWC (%)	BD (g cm^−3^)	PR (MPa)
0–15 cm	Emergence	Conventional control	42.2 Aa ^ns^	1.28 Aa ^ns^	0.84 Aa *
		Conventional manure	43.8 Aa ^ns^	1.31 Aa *	0.82 Aa ^ns^
		Conservation control	40.4 Aa ^ns^	1.30 Aa ^ns^	0.89 Aa ^ns^
		Conservation manure	41.6 Aa ^ns^	1.27 Aa ^ns^	0.64 Aa *
	Harvest	Conventional control	41.7 Aa ^ns^	1.31 Aa ^ns^	0.78 Aa ^ns^
		Conventional manure	41.4 Aa ^ns^	1.34 Aa ^ns^	0.72 Aa *
		Conservation control	42.6 Aa ^ns^	1.34 Aa ^ns^	1.10 Aa ^ns^
		Conservation manure	39.9 Aa ^ns^	1.29 Aa ^ns^	0.73 Aa ^ns^
15–30 cm	Emergence	Conventional control	39.8 Aa ^ns^	1.35 Aa ^ns^	1.45 Aa *
		Conventional manure	41.9 Aa ^ns^	1.38 Aa *	1.07 Aa ^ns^
		Conservation control	39.5 Aa ^ns^	1.32 Aa ^ns^	1.19 Aa ^ns^
		Conservation manure	41.7 Aa ^ns^	1.31 Aa ^ns^	1.10 Aa *
	Harvest	Conventional control	38.6 Aa ^ns^	1.36 Aab ^ns^	1.17 Aa ^ns^
		Conventional manure	40.8 Aa ^ns^	1.39 Aa ^ns^	1.23 Aa *
		Conservation control	39.5 Aa ^ns^	1.32 Ab ^ns^	1.47 Aa ^ns^
		Conservation manure	39.87 Aa ^ns^	1.33 Ab ^ns^	1.08 Aa ^ns^

**Table 2:** Results of two-way ANOVA analysis considering soil properties. Different letters after mean values in the columns represent significant differences at *p* < 0.05. Capital letters show statistical differences between treatments in one season; small letters show statistical differences for treatment between two seasons. Abbreviations: WSA, water-stable aggregates; SOM, soil organic matter. Data were collected in May (emergence) and October (harvest) 2021 at a field close to Marija Magdalena, northwestern Croatia.

Time	Treatment	WSA	SOM (%)
Emergence	Conventional control	62.9 Aa	3.47 Aa
	Conventional manure	50.4 Ab	3.35 Aa
	Conservation control	52.4 Ab	3.43 Aa
	Conservation manure	61.4 Aa	3.38 Aa
Harvest	Conventional control	49.3 Bb	3.12 Ba
	Conventional manure	67.9 ABa	3.77 Aa
	Conservation control	63.9 ABa	3.47 ABa
	Conservation manure	73.9 Aa	3.46 ABa

**Table 3:** Soil properties at 0–35 cm (Ap horizon) and 35–70 cm (Btg horizon) depths.

Soil Properties	0–35 cm	35–70 cm
pH in H_2_O	7.29	5.10
P_2_O_5_ (mg kg^−1^)	163	42
K_2_O (mg kg^−1^)	282	79
Organic matter (%)	3.37	1.9
Bulk density (g cm^−3^)	1.26	1.34
Water holding capacity (%)	44.0	30.7
Clay (%)	23.2	39.9
Silt (%)	30.4	21
Sand (%)	46.4	39.1
Texture	Loam	Clay Loam

**Table 4:** Characteristics of the tractor and equipment used during the trial.

Operation	Tractor Used	Equipment Used	Mass (kg)
Ploughing	SAME Antares 100	Vogel & Noot—Farmer L950	3610 + 225
Vertical loosening	SAME Antares 100	Lemken-Achat 70-3/9	3610 + 440
Seedbed preparation	SAME Antares 100	MASCHIO DRAGO DC	3610 + 770
Sowing	SAME Atlanta 45	OLT MSK-4	1840 + 480
Herbicide application	SAME Atlanta 45	Tolmet Klara 412-3	1840 + 170
Foliar feeding	SAME Atlanta 45	Tolmet Klara 412-3	1840 + 170
Harvest	Deutz-Fahr Topliner 4060		9255
Chopping harvest residues	SAME Atlanta 45	PANEX AGM-145	1840 + 270

## Data Availability

The data presented in this study are available on reasonable request from the corresponding author.

## References

[1] Lal R. (2020). Soil organic matter content and crop yield. J. Soil Water Conserv..

[2] Guo Q., Hao Y., Liu B. (2015). Rates of soil erosion in China: A study based on runoff plot data. Catena.

[3] Pimentel D., Burgess M. (2013). Soil erosion threatens food production. Agriculture.

[4] Kisic I., Bogunovic I., Birkás M., Jurisic A., Spalevic V. (2017). The role of tillage and crops on a soil loss of an arable Stagnic Luvisol. Arch. Agron. Soil Sci..

[5] Alcántara V., Don A., Well R., Nieder R. (2016). Deep ploughing increases agricultural soil organic matter stocks. Glob. Chang. Biol..

[6] Bogunovic I., Pereira P., Kisic I., Sajko K., Sraka M. (2018). Tillage management impacts on soil compaction, erosion and crop yield in Stagnosols (Croatia). Catena.

[7] Pagliai M., Vignozzi N., Pellegrini S. (2004). Soil structure and the effect of management practices. Soil Till. Res..

[8] Vian J.F., Peigné J., Chaussod R., Roger-Estrade J. (2009). Effects of four tillage systems on soil structure and soil microbial biomass in organic farming. Soil Use Manag..

[9] Tromp-van Meerveld H.J., Parlange J.Y., Barry D.A., Tromp M.F., Sander G.C., Walter M.T., Parlange M.B. (2008). Influence of sediment settling velocity on mechanistic soil erosion modeling. Water Resour. Res..

[10] Bronick C.J., Lal R. (2005). Soil structure and management: A review. Geoderma.

[11] Shokati B., Ahangar A.G. (2014). Effect of conservation tillage on soil fertility factors: A review. Int. J. Biosci..

[12] Karlen D.L., Kovar J.L., Cambardella C.A., Colvin T.S. (2013). Thirty-year tillage effects on crop yield and soil fertility indicators. Soil Till. Res..

[13] Seitz S., Goebes P., Puerta V.L., Pereira E.I.P., Wittwer R., Six J., van der Heijden M.G.A., Scholten T. (2019). Conservation tillage and organic farming reduce soil erosion. Agron. Sustain. Dev..

[14] Gholami L., Sadeghi S.H.R., Homaee M. (2016). Different effects of sheep manure conditioner on runoff and soil loss components in eroded soil. Catena.

[15] Bogunović I., Hrelja I., Kisić I., Dugan I., Krevh V., Defterdarović J., Filipović V., Filipović L., Pereira P. (2023). Straw Mulch Effect on Soil and Water Loss in Different Growth Phases of Maize Sown on Stagnosols in Croatia. Land.

[16] Mohammed S., Hassan E., Abdo H.G., Szabo S., Mokhtar A., Alsafadi K., Rodrigo-Comino J. (2021). Impacts of rainstorms on soil erosion and organic matter for different cover crop systems in the western coast agricultural region of Syria. Soil Use Manag..

[17] Amini S., Asoodar M.A. (2015). Investigation on the effect of conservation tillage on soil organic matter (SOM) and soil organic carbon (SOC): The review. N. Y. Sci. J..

[18] Li H., Gao H., Wu H., Li W., Wang X., He J. (2007). Effects of 15 years of conservation tillage on soil structure and productivity of wheat cultivation in northern China. Soil Res..

[19] Puerta V.L., Pereira E.I.P., Wittwer R., Van Der Heijden M., Six J. (2018). Improvement of soil structure through organic crop management, conservation tillage and grass-clover ley. Soil Till. Res..

[20] Blanco-Canqui H., Lal R. (2009). Crop residue removal impacts on soil productivity and environmental quality. Crit. Rev. Plant Sci..

[21] Van Donk S.J., Martin D.L., Irmak S., Melvin S.R., Petersen J.L., Davison D.R. (2010). Crop residue cover effects on evaporation, soil water content, and yield of deficit-irrigated corn in west-central Nebraska. Trans. ASABE.

[22] Salinas-Garcia J.R., Hons F.M., Matocha J.E. (1997). Long-term effects of tillage and fertilization on soil organic matter dynamics. Soil Sci. Soc. Am. J..

[23] Karlen D.L., Wollenhaupt N.C., Erbach D.C., Berry E.C., Swan J.B., Eash N.S., Jordahl J.L. (1994). Long-term tillage effects on soil quality. Soil Till. Res..

[24] Bessam F., Mrabet R. (2003). Long-term changes in soil organic matter under conventional tillage and no-tillage systems in semiarid Morocco. Soil Use Manag..

[25] Büchi L., Wendling M., Amossé C., Jeangros B., Sinaj S., Charles R. (2017). Long and short term changes in crop yield and soil properties induced by the reduction of soil tillage in a long term experiment in Switzerland. Soil Till. Res..

[26] Rice C.W. (2002). Organic matter and nutrient dynamics. Encyclopedia of Soil Science.

[27] Zingore S., Delve R.J., Nyamangara J., Giller K.E. (2008). Multiple benefits of manure: The key to maintenance of soil fertility and restoration of depleted sandy soils on African smallholder farms. Nutr. Cycl. Agroecosyst..

[28] Larney F.J., Hao X., Topp E. (2011). Manure management. Soil Management: Building a Stable Base for Agriculture.

[29] Riley H., Pommeresche R., Eltun R., Hansen S., Korsaeth A. (2008). Soil structure, organic matter and earthworm activity in a comparison of cropping systems with contrasting tillage, rotations, fertilizer levels and manure use. Agric. Ecosyst. Environ..

[30] de Melo T.R., Figueiredo A., Machado W., Tavares Filho J. (2019). Changes on soil structural stability after in natura and composted chicken manure application. Int. J. Recycl. Org. Waste Agric..

[31] Panagos P., Montanarella L., Barbero M., Schneegans A., Aguglia L., Jones A. (2022). Soil priorities in the European Union. Geoderma Reg..

[32] Heuser I. (2022). Soil Governance in current European Union Law and in the European Green Deal. Soil Secur..

[33] Gicheru P., Gachene C., Mbuvi J., Mare E. (2004). Effects of soil management practices and tillage systems on surface soil water conservation and crust formation on a sandy loam in semi-arid Kenya. Soil Till. Res..

[34] Goldberg N., Nachshon U., Argaman E., Ben-Hur M. (2020). Short term effects of livestock manures on soil structure stability, runoff and soil erosion in semi-arid soils under simulated rainfall. Geosciences.

[35] Martín-Lammerding D., Gabriel J.L., Zambrana E., Santín-Montanyá I., Tenorio J.L. (2021). Organic amendment vs. Mineral fertilization under minimum tillage: Changes in soil nutrients, soil organic matter, biological properties and yield after 10 years. Agriculture.

[36] Materechera S.A. (2009). Tillage and tractor traffic effects on soil compaction in horticultural fields used for peri-urban agriculture in a semi-arid environment of the North West Province, South Africa. Soil Till. Res..

[37] Badalíková B. (2010). Influence of soil tillage on soil compaction. Soil Engineering.

[38] Sidhu D., Duiker S.W. (2006). Soil compaction in conservation tillage: Crop impacts. Agron. J..

[39] Afzalinia S., Zabihi J. (2014). Soil compaction variation during corn growing season under conservation tillage. Soil Till. Res..

[40] Jakab G., Madarász B., Szabó J.A., Tóth A., Zacháry D., Szalai Z., Kertesz A., Dyson J. (2017). Infiltration and soil loss changes during the growing season under ploughing and conservation tillage. Sustainability.

[41] Fleming R.L., Powers R.F., Foster N.W., Kranabetter J.M., Scott D.A., Ponder F., Berch S., Chapman W.K., Kabzems R.D., Ludovici K.H. (2006). Effects of organic matter removal, soil compaction, and vegetation control on 5-year seedling performance: A regional comparison of Long-Term Soil Productivity sites. Can. J. For. Res..

[42] De Neve S., Hofman G. (2000). Influence of soil compaction on carbon and nitrogen mineralization of soil organic matter and crop residues. Biol. Fertil. Soils.

[43] Franzluebbers A.J. (2002). Water infiltration and soil structure related to organic matter and its stratification with depth. Soil Till. Res..

[44] Lampurlanés J., Cantero-Martínez C. (2003). Soil bulk density and penetration resistance under different tillage and crop management systems and their relationship with barley root growth. Agron. J..

[45] Schlüter S., Großmann C., Diel J., Wu G.M., Tischer S., Deubel A., Rücknagel J. (2018). Long-term effects of conventional and reduced tillage on soil structure, soil ecological and soil hydraulic properties. Geoderma.

[46] Subbulakshmi S., Saravanan N., Subbian P. (2009). Conventional tillage vs conservation tillage—A review. Agric. Rev..

[47] Jeřábek J., Zumr D., Dostál T. (2017). Identifying the plough pan position on cultivated soils by measurements of electrical resistivity and penetration resistance. Soil Till. Res..

[48] Wagner S., Cattle S.R., Scholten T. (2007). Soil-aggregate formation as influenced by clay content and organic-matter amendment. J. Plant Nutr. Soil Sci..

[49] Birkás M., Szemők A., Antos G., Neményi M. (2008). Environmentally-Sound Adaptable Tillage.

[50] Paustian K., Six J., Elliott E.T., Hunt H.W. (2000). Management options for reducing CO_2_ emissions from agricultural soils. Biogeochemistry.

[51] Mikha M.M., Rice C.W. (2004). Tillage and manure effects on soil and aggregate-associated carbon and nitrogen. Soil Sci. Soc. Am. J..

[52] Six J., Elliott E.T., Paustian K. (2000). Soil structure and soil organic matter II. A normalized stability index and the effect of mineralogy. Soil Sci. Soc. Am. J..

[53] Haynes R.J. (2005). Labile organic matter fractions as centralcomponents of the quality of agricultural soils: An overview. Adv. Agron..

[54] Lin Y., Ye G., Kuzyakov Y., Liu D., Fan J., Ding W. (2019). Long-term manure application increases soil organic matter and aggregation, and alters microbial community structure and keystone taxa. Soil Biol. Biochem..

[55] Mohammadi K., Heidari G., Khalesro S., Sohrabi Y. (2011). Soil management, microorganisms and organic matter interactions: A review. Afr. J. Biotechnol..

[56] Turmel M.S., Speratti A., Baudron F., Verhulst N., Govaerts B. (2015). Crop residue management and soil health: A systems analysis. Agric. Syst..

[57] Congreves K.A., Hayes A., Verhallen E.A., Van Eerd L.L. (2015). Long-term impact of tillage and crop rotation on soil health at four temperate agroecosystems. Soil Till. Res..

[58] Kihara J., Bationo A., Mugendi D.N., Martius C., Vlek P.L. (2011). Conservation tillage, local organic resources, and nitrogen fertilizer combinations affect maize productivity, soil structure and nutrient balances in semi-arid Kenya. Innovations as Key to the Green Revolution in Africa: Exploring the Scientific Facts.

[59] Busari M.A., Kukal S.S., Kaur A., Bhatt R., Dulazi A.A. (2015). Conservation tillage impacts on soil, crop and the environment. Int. Soil Water Conserv. Res..

[60] Govaerts B., Sayre K.D., Goudeseune B., De Corte P., Lichter K., Dendooven L., Deckers J. (2009). Conservation agriculture as a sustainable option for the central Mexican highlands. Soil Till. Res..

[61] Neave M., Rayburg S. (2007). A field investigation into the effects of progressive rainfall-induced soil seal and crust development on runoff and erosion rates: The impact of surface cover. Geomorphology.

[62] Pingping H., Xue S., Li P., Zhanbin L. (2013). Effect of vegetation cover types on soil infiltration under simulating rainfall. Nat. Environ. Pollut. Technol..

[63] Leys A., Govers G., Gillijns K., Berckmoes E., Takken I. (2010). Scale effects on runoff and erosion losses from arable land under conservation and conventional tillage: The role of residue cover. J. Hydrol..

[64] Raczkowski C.W., Reyes M.R., Reddy G.B., Busscher W.J., Bauer P.J. (2009). Comparison of conventional and no-tillage corn and soybean production on runoff and erosion in the southeastern US Piedmont. J. Soil Water Conserv..

[65] Roose E., Barthes B. (2001). Organic matter management for soil conservation and productivity restoration in Africa: A contribution from Francophone research. Managing Organic Matter in Tropical Soils: Scope and Limitations: Proceedings of a Workshop organized by the Center for Development Research at the University of Bonn (ZEF Bonn)—Germany, 7–10 June, 1999.

[66] Arriaga F.J., Lowery B. (2003). Soil physical properties and crop productivity of an eroded soil amended with cattle manure. Soil Sci..

[67] Gilley J.E., Risse L.M. (2000). Runoff and soil loss as affected by the application of manure. Trans. ASAE.

[68] Gabriel J.L., García-González I., Quemada M., Martin-Lammerding D., Alonso-Ayuso M., Hontoria C. (2021). Cover crops reduce soil resistance to penetration by preserving soil surface water content. Geoderma.

[69] Hamza M.A., Anderson W.K. (2005). Soil compaction in cropping systems: A review of the nature, causes and possible solutions. Soil Till. Res..

[70] Aziz T., Ullah S., Sattar A., Nasim M., Farooq M., Khan M.M. (2010). Nutrient availability and maize (*Zea mays*) growth in soil amended with organic manures. Int. J. Agric. Biol..

[71] Chadwick D., Wei J., Yan’an T., Guanghui Y., Qirong S., Qing C. (2015). Improving manure nutrient management towards sustainable agricultural intensification in China. Agric. Ecosyst. Environ..

[72] Cai A., Xu M., Wang B., Zhang W., Liang G., Hou E., Luo Y. (2019). Manure acts as a better fertilizer for increasing crop yields than synthetic fertilizer does by improving soil fertility. Soil Till. Res..

[73] Gathala M.K., Timsina J., Islam M.S., Rahman M.M., Hossain M.I., Harun-Ar-Rashid M., Ghosh A.K., Krupnik T.J., Tiwari T.P., McDonald A. (2015). Conservation agriculture based tillage and crop establishment options can maintain farmers’ yields and increase profits in South Asia’s rice–maize systems: Evidence from Bangladesh. Field Crops Res..

[74] Vogeler I., Rogasik J., Funder U., Panten K., Schnug E. (2009). Effect of tillage systems and P-fertilization on soil physical and chemical properties, crop yield and nutrient uptake. Soil Till. Res..

[75] Crittenden S.J., Poot N., Heinen M.D.J.M., Van Balen D.J.M., Pulleman M.M. (2015). Soil physical quality in contrasting tillage systems in organic and conventional farming. Soil Till. Res..

[76] Pittelkow C.M., Linquist B.A., Lundy M.E., Liang X., Van Groenigen K.J., Lee J., van Gestel N., Six J., Venterea R.T., Van Kessel C. (2015). When does no-till yield more? A global meta-analysis. Field Crops Res..

[77] Husnjak S., Filipovic D., Kosutic S. (2002). Influence of different tillage systems on soil physical properties and crop yield. Rostlinna Vyroba.

[78] Xiao-Bin W., Dian-Xiong C.A.I., Hoogmoed W.B., Oenema O., Perdok U.D. (2006). Potential effect of conservation tillage on sustainable land use: A review of global long-term studies. Pedosphere.

[79] Lampurlanés J., Plaza-Bonilla D., Álvaro-Fuentes J., Cantero-Martínez C. (2016). Long-term analysis of soil water conservation and crop yield under different tillage systems in Mediterranean rainfed conditions. Field Crops Res..

[80] Xu J., Han H., Ning T., Li Z., Lal R. (2019). Long-term effects of tillage and straw management on soil organic carbon, crop yield, and yield stability in a wheat-maize system. Field Crops Res..

[81] Rusinamhodzi L., Corbeels M., Van Wijk M.T., Rufino M.C., Nyamangara J., Giller K.E. (2011). A meta-analysis of long-term effects of conservation agriculture on maize grain yield under rain-fed conditions. Agron. Sustain. Dev..

[82] Maguire R.O., Kleinman P.J., Dell C.J., Beegle D.B., Brandt R.C., McGrath J.M., Ketterings Q.M. (2011). Manure application technology in reduced tillage and forage systems: A review. J. Environ. Qual..

[83] Van Apeldoorn D.F., Sonneveld M.P.W., Kok K. (2011). Landscape asymmetry of soil organic matter as a source of agro-ecosystem resilience. Agric. Ecosyst. Environ..

[84] Lal R. (2016). Soil health and carbon management. Food Energy Secur..

[85] Allen D.E., Singh B.P., Dalal R.C. (2011). Soil health indicators under climate change: A review of current knowledge. Soil Health and Climate Change.

[86] Kottek M., Grieser J., Beck C., Rudolf B., Rubel F. (2016). World map of the Köppen-Geiger climate classification updated. Meteorol. Z..

[87] IUSS—WRB (2014). World Reference Base for Soil Resources 2014: International Soil Classification System for Naming Soils and Creating Legends for Soil Maps.

[88] Bogunovic I., Telak L.J., Pereira P. (2020). Experimental comparison of runoff generation and initial soil erosion between vineyards and croplands of Eastern Croatia: A case study. Air Soil Water Res..

[89] Schindewolf M., Schmidt J. (2012). Parameterization of the EROSION 2D/3D soil erosion model using a small-scale rainfall simulator and upstream runoff simulation. Catena.

[90] Dıaz-Zorita M., Perfect E., Grove J.H. (2002). Disruptive methods for assessing soil structure. Soil Till. Res..

[91] Kemper W.D., Rosenau R.C., Klute A. (1986). Aggregate stability and size distribution. Methods of Soil Analysis.

[92] Walkley A., Black I.A. (1934). An examination of the Digestion method for determining soil organic matter, and a proposed modification of the chromic acid titration method. Soil Sci..

[93] Statsoft (2015). Statistica 12.0 Software.

[94] Plotly Chart Studio. https://chart-studio.plotly.com/.

